# Donor Safety in Living Donor Liver Transplantation: A Single-Center Analysis of 300 Cases

**DOI:** 10.1371/journal.pone.0061769

**Published:** 2013-04-25

**Authors:** Jianyong Lei, Lunan Yan, Wentao Wang

**Affiliations:** Departments of Liver and Vascular Surgery, West China Hospital of Sichuan University, Chengdu, China; Beijing Institute of Infectious Diseases, China

## Abstract

**Aim:**

To evaluate the safety to donors of living-donor liver transplantation.

**Methods:**

This study included 300 consecutive living liver tissue donors who underwent operations at our center from July 2002 to December 2012. We evaluated the safety of donors with regard to three aspects complications were recorded prospectively and stratified by grade according to Clavien’s classification, and the data were compared in two stages (the first 5 years’ experience (pre-January 2008) and the latter 5 years’ experience (post-January 2008); laboratory tests such as liver function and blood biochemistry were performed; and the health-related quality of life was evaluated.

**Results:**

There was no donor mortality at our center, and the overall morbidity rate was 25.3%. Most of the complications of living donors were either grade I or II. There were significantly fewer complications in the latter period of our study than in the initial period (19.9% *vs* 32.6%, *P*<0.001), and biliary complications were the most common complications, with an incidence of 9%. All of the liver dysfunction was temporary; however, the post-operative suppression of platelet count lasted for years. Although within the normal range, eight years after operation, 22 donors showed lower platelet levels (189×10^9^/L) compared with the pre-operative levels (267×10^9^/L) (*P<*0.05). A total of 98.4% of donors had returned to their previous levels of social activity and work, and 99.2% of donors would donate again if it was required and feasible. With the exception of two donors who experienced grade III complications (whose recipients died) and a few cases of abdominal discomfort, fatigue, chronic pain and scar itching, none of the living donors were affected by physical problems.

**Conclusion:**

With careful donor selection and specialized patient care, low morbidity rates and satisfactory long-term recovery can be achieved after hepatectomy for living-donor liver transplantation.

## Introduction

Transplantation is the exclusive treatment of choice for patients with end-stage liver disease. However, the increasing rates of death, especially in China, where there are a large number of hepatitis B virus (HBV)infected patients and the brain death law is not accepted by the public because of social customs, have led to the increasing use of the relatively risky approach of living-donor liver transplantation (LDLT). The first LDLTs were performed in pediatric recipients in 1989 [1], and the first successful LDLT in an adult recipient was performed by Haberal *et al* [2] in 1992. The first such operation in mainland China occurred in 2002 [3], and LDLT has evolved dramatically over the past decade. This procedure has been criticized for the risk, including potential death, that it imposes on healthy individuals who will undergo a major operation without any potential health benefit. After several living-donor deaths were reported in the United States and in Europe[4]–[6], increasingly cautious approaches to this procedure have been adopted [7]. Donor morbidity ranges from 9.4% to 75%[8]–[14]. Almost all donors experience short-term liver dysfunction and routine blood count abnormalities [13], [15]–[17]. Although laboratory test results may guide surgery and identify complications earlier, some complications may lead to physical, mental, and psychosocial problems that affect the quality of life and psychological outcomes of living donors after transplantation. Therefore, it is important to precisely evaluate the surgical complications, liver dysfunction and quality of life of living donors after operation.

With increasing numbers of LDLTs being performed, there is an increasing concern about the safety of living liver donors; however, no systematic and sufficiently large reports on this subject are available, and few authors have considered quality of life measures in addition to surgical and laboratory complications [6], [14], [18]. In the present study, we analyzed the first 300 consecutive living-donor operations since the inception of our program in 2002. We compared two different periods (the first 5 years and the latter 5 years) in terms of the standardized classification of severity of complications, including an evaluation of liver function and health-related quality of life.

## Materials and Methods

From July 2002 to December 2012, we performed 300 LDLTs (including 250 LRLTs without a middle hepatic vein (MHV), one LRLT with MHV, seven with a left-lateral lobe at segments II and III, and 42 with a left lobe at segment II-IV) at the West China Hospital. We reviewed the donors’ demographics, operative details, and post-operative complications, which were recorded according to the Clavien classification. Short- and long-term changes in liver function, including total bilirubin, aspartate aminotransferase (AST), alanine aminotransferase (ALT), international rate (INR), albumin (ALB) and the volume of ascites, were recorded. A long-term follow-up of routine blood parameters was performed, including white blood cell (WBC) count, platelet (PLT) count and red blood cell (RBC) count. Health-related quality-of-life (HRQoL) measures, including the physical health, mental health, and vocational impact of the donors, were as assessed with the Chinese Version of the Medical Outcomes Study Short Form-36 tool [19]. Psychological symptoms were measured using the Symptom Checklist-90-Revised (SCL-90-R) [20]. HRQoL measures and the psychological outcome data were obtained from the questionnaires. The relevant clinical data, in particular the complications of the living donors, were compared for cases performed before (first 5 years’ experience, group 1, *n* = 129) and after January 2008 (latter 5 years’ experience, group 2, *n* = 154).

### Donor Selection and Evaluation

The transplant selection process included an initial evaluation that consisted of health screening, blood tests, viral serology, imaging studies and medical and psychiatric assessment. All of the data were collected from the Chinese Liver Transplant Registry: http://www.cltr.org. The inclusion and exclusion criteria used here have been previously described [21]. We informed the donors and their families of the possible risks of donor hepatectomy. Multi-row-detector computerized tomographic (CT) scans for volumetric measurements were performed to evaluate graft size, hepatic vascular anatomy (including hepatic artery, portal vein, and hepatic vein), and remaining donor liver size, and magnetic resonance imaging was used to evaluate the biliary tract. Steatosis of the liver was assessed by CT and the calculation of body mass index instead of a biopsy [22]. Fibroscan or elastography was used in individual donors to detect liver steatosis or fibrosis preoperatively and for intraoperative evaluation of liver quality. The donor must have had three or fewer degrees of consanguinity with the recipient, as verified by the Health Administrative Departments and the Public Security Organs or by a DNA test. All procedures were performed after approval from the Ethics Committee of Sichuan University and the local authority was obtained. In addition, the donation was voluntary and altruistic. Meanwhile, we informed the donors and their families of the possible risks of donor hepatectomy. The donors gave written consent for their information to be stored in the hospital database and used for research.

### Surgical Technique

We dissected the right or left hepatic artery and the right or left portal vein, defined the hepatic venous drainage of the right or left liver lobe using intraoperative ultrasound, and then isolated the hepatic vein. Next, we divided the attachments between the right or left lobe and the diaphragm to expose the inferior right or left hepatic veins. The right or left bile duct was then cut sharply. Without hepatic vascular occlusion, the hepatic parenchyma was separated with a Cavitron Ultra-Sonic Aspirator (CUSA EXcel, Valleylab, Boulder, CO, United States) and an argon knife. When the graft was completely separated, we quickly removed the graft to a table and flushed it with University of Wisconsin solution through the pulmonary veins (PV) and hepatic artery at 4°C. The volume of the grafts was measured with a 3-L beaker using a drainage method intraoperatively, and the error was within 10 mL [23], [24]. Donors stayed in the intensive care unit (ICU) for the day of the operation and were transferred to the surgical ward when their conditions stabilized.

### Statistical Analysis

Descriptive data are expressed as medians with standard deviations (SD). Continuous variables were compared as independent samples using the nonparametric Wilcoxon test because some of the measurements did not follow a normal distribution. Categorical data were compared using the chi-squared test or Fisher’s test when appropriate. Inclusion of variables into the final models was based on both biological and statistical considerations. SPSS version 17.0 was used for all data management and statistical analyses. *P<*0.05 was considered statistically significant.

## Results

### Donor Characteristics

All donors were alive and well at the end of follow-up, which averaged 45 months (range, 3–130 months). The patient characteristics are presented in Table 1. Right hepatectomy (without MHV), left hepatectomy, and left lateral segmentectomy were performed in 251, 42 and 7 donors, respectively, and one donor underwent right hepatectomy with MHV for the small graft. There were relatively more left and left lateral donors before January 2008 than after because most of our initial recipients were children. The overall mean age of donors was 35.4 years (range, 19–62 years), and the mean ages of groups 1 and 2 were 36.2 and 34.9 years, respectively (*P*>0.05). There was no difference between the two groups in the donors’ gender, weight (61.1 kg *vs* 61.7 kg), height (166.8 cm *vs* 163.3 cm), body mass index (22.7 kg/m^2^ *vs* 23.3 kg/m^2^) or relationships with the recipients.

**Table 1:** Donor Characteristics, Operative Data and Outcomes of Donors: Comparisons between Our Two Stages of Experience (Pre-January 2008 and Post- January 2008).

	Total number	Pre-2008 group	Post-2008 group	P Value
**Donor characteristics**				
Donor number	300	129	171	NS
Right hepatectomy (without MHV)	250	105	145	
Right hepatectomy (with MHV)	1	0	1	
Left hepatectomy	42	21	21	
Left lateral hepatectomy	7	3	4	
Age (yr), mean±SD		36.2±7.8	34.9±11.2	NS
Gender (M:F)		73: 56	98: 73	NS
Weight (kg), mean±SD		61.1±8.9	61.7±10.2	NS
Height (cm), mean±SD		166.8±8.2	163.3±7.7	NS
BMI (kg/m^2^), mean±SD		22.7±2.6	23.3±3.0	NS
HBsAb (+/−)	136/164	62/77	74/87	NS
HBcAb (+/−)	20/280	9/120	11/160	NS
Relationship to recipient				NS
Parent	71	28	43	
Child	38	17	21	
Spouse	99	46	53	
Sibling	45	18	27	
Other relative	47	20	27	
**Intra-operative data**				
Graft size (g)		533.8±166.9	501.6±143.3	NS
Graft/donor weight (GRWR)		0.88±0.43	0.85±0.49	NS
Operative time (h)		7.4±1.6	6.0±1.1	P<0.05
Blood loss (mL)		698.1±559.2	561.2±573.1	P<0.05
Autologous blood transfusion (mL)		393.3±196.1	344.2±234.8	NS
Rate of allogeneic transfusion		13.9	15.8	NS
**Outcomes of donors**				
ICU stay (d)		2.11±0.33	2.21±0.56	NS
Hospital stay (d)		12.8±4.6	8.7±3.3	P<0.05
Cost of hospitalization (US dollars)		4058.1±376.3	5441.6±781.4	P<0.05
Clavien score				
0	224	87	137	P<0.05
1	39	22(17.1%)	17(9.9%)	P<0.05
2	19	9(7.0%)	10(5.8%)	NS
3	17	11(8.5%)	6(3.5%)	P<0.05
4	1	0	1(0.6%)	NS

MHV: middle hepatic vein; SD: standard deviation; HBsAb: hepatitis B surface antibody; HBcAb: hepatitis B core antibody; BMI: body mass index; NS: not significant.

### Operative Details and Outcomes

The intra-operative data of all the donors were collected retrospectively. The differences between the two groups are compared in Table 1. There were no significant differences between the two groups in the graft size, graft/recipient weight ratio (GRWR), autologous blood transfusions, or length of ICU stay; however, significant differences in operative time, blood loss, length of hospital stay, and the cost of the hospitalization were observed (P<0.05 for each). There was less mean blood loss (561.2 mL) after January 2008 than before (698.1 mL), while the operation time was also shortened to 6.0 h in the second stage compared to 7.4 h in the first stage. These improvements were attributable to the improved techniques used in surgery and anesthesia after January 2008. Interestingly, the second stage had a higher average hospitalization cost of 5441.6 US dollars compared with 4058.1 US dollars in the first stage (*P<*0.05). This difference was mainly caused by the persistent rise in commodity prices in China in recent years.

### Donor Complications

One donor had an intra-operative complication (massive hemorrhage from a ruptured right hepatic vein), and this emergency was controlled by suturing the vein. The post-operative complications classified according to the Clavien system are shown in Tables 1 and 2, and the overall complication rate was 25.3%, of which half (51.3%) were minor complications (grade 1). As shown in Table 2, the most common complications were biliary complications, including biliary leak and biliary stricture, with an incidence of 9%. Twenty-two donors (7.3%) suffered infection after donation. The most common site for infection was the surgical wound, followed by the abdominal cavity. No significant difference was observed in the infection rate between the two groups. Although only 8 patients suffered postoperative bleeding, all of them needed therapy, and 6 needed reoperation. However, most patients (76.9%) who suffered effusion did not need any therapy. Only one donor was diagnosed with portal vein thrombosis, and this patient underwent portal vein embolectomy. Before **January** 2008, the complication rate was 32.6%, which was much higher than the 19.9% in the second stage. The severity of the complications was also much higher in the first stage than in the second stage. No grade 5 complication (donor morbidity) was observed in either stage. A 65-year-old donor in the second stage suffered liver failure after donating 581 g (45.1%) of the right lobe of the liver (without MHV). The donor’s TB increased to 388.2 µmol/L 16 days after donation, the 24 h volume of ascites was approximately 1100 mL, and he suffered hepatic encephalopathy (grade II) after donation, but after positive therapies (artificial liver replacement therapy and medication), he recovered well and was discharged from our hospital 34 days after operation. After 4 months’ follow-up, this donor’s status was good. We think the main reason for his complications was the difference between his stated age and actual age: the patient, his family, and his identity card indicated he was 58 years old in 2012, but he was actually 65 years old, as his family revealed after this serious adverse event.

**Table 2:** Comparisons of Complications between the Two Groups.

Complications	Clavien grade 1		Clavien grade 2		Clavien grade 3	
	pre-2008	post-2008	pre-2008	post-2008	pre-2008	post-2008
Overall complications	22(17.1%)	17(9.9%)	9(7.0%)	10(5.8%)	11(8.5%)	6(3.5%)
Biliary complications	7(5.4%)	6(3.5%)	4(3.1%)	4(2.3%)	4(3.1%)	2(1.2%)
Biliary leak	7	6	4	4	2	1
Biliary stricture	0	0	0	0	2	1
Infection	6(4.7%)	7(4.1%)	2(1.6%)	2(1.2%)	3(2.3%)	2(1.2%)
Wound infection	2	5	1	2	1	1
Intraabdominal infection	4	2	0	0	2	1
Lung	0	0	1	0	0	0
Postoperative bleeding	0	0	2(1.6%)	3(1.8%)	1(0.8%)	2(1.2%)
Intraabdominal bleeding	0	0	1	2	1	2
Incision bleeding	0	0	1	1	0	0
Effusion	6 (4.7%)	4 (2.3%)	1 (0.8%)	1 (0.6%)	1 (0.8%)	0
Pleural effusion	2	2	1	1	1	0
Intraabdominal effusion	4	2	0	0	0	0
Vascular complications						
Portal vein thrombosis	0	0	0	0	1	0
Others	3	0	0	0	1	0

Others: voice change, lymph leakage, hepatic encephalopathy.

### Laboratory Value Changes after Donation

**Figure 1:**
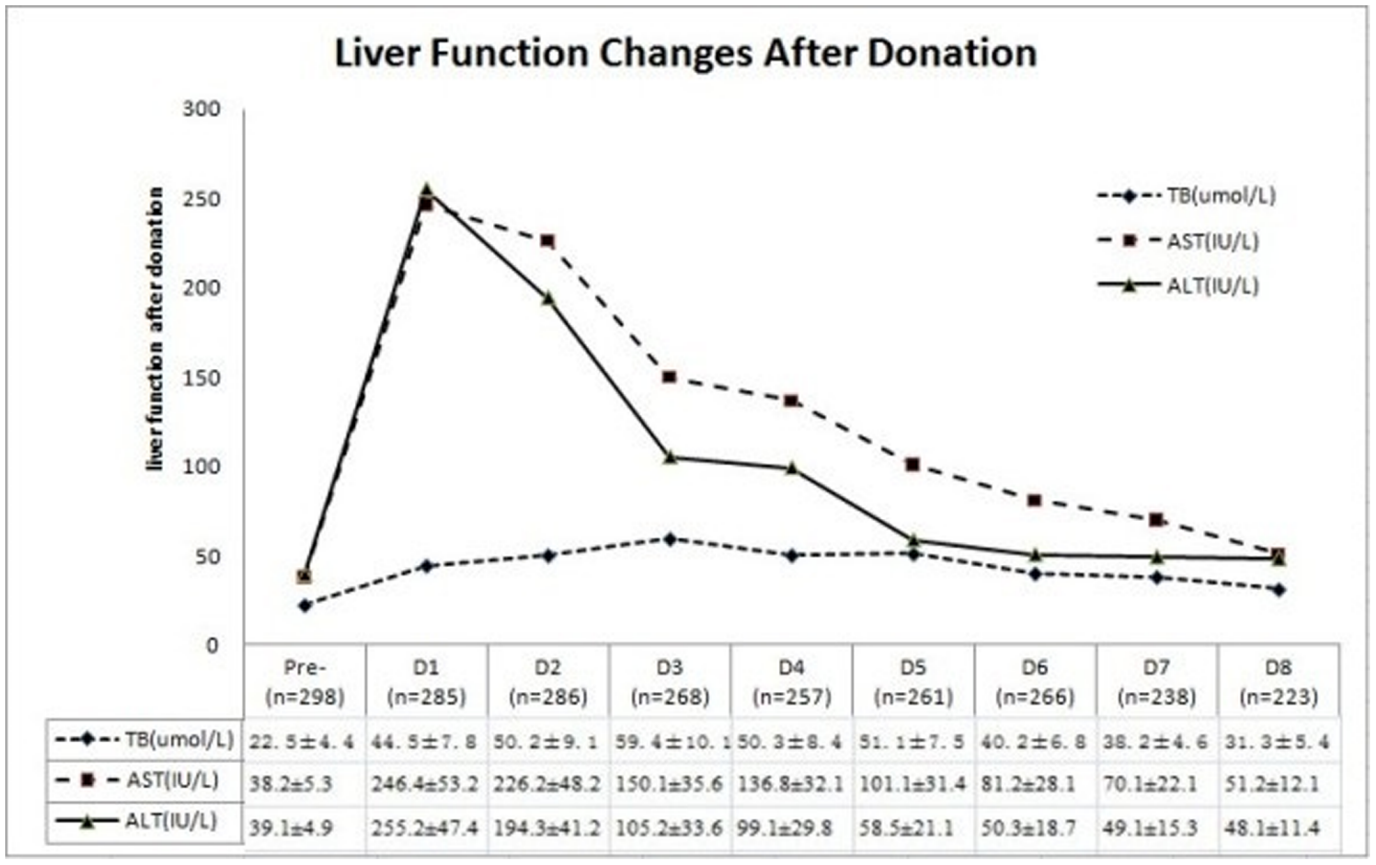
Changes in liver function (total bilirubin, aspartate aminotransferase, alanine aminotransferase) after donation.

After harvesting, donor liver functions were impaired to different degrees, including transient liver enzyme elevation, hyperbilirubinemia, and hypoalbuminemia during the immediate postoperative period (first several days). Similar abnormalities were observed in prothrombin time; however, most of these indices gradually returned to normal within 30 d after the operation. AST and ALT reached their peaks on the first post-operative day, while the TB reached its peak on the 3^rd^ post-operative day; however, almost all returned to normal within 30 d after conservative treatment (Figure 1). The changes in AST, ALT and TB after operation are shown in Figure 1. The mean volumes of ascites were 125±66 mL on the first day and 67±45 mL on the second day, and most of the drainage tubes were extracted on post-operative days 3–4, with only 3 donors’ tubes being left longer than 5 d, due to biliary leaks.

**Figure 2:**
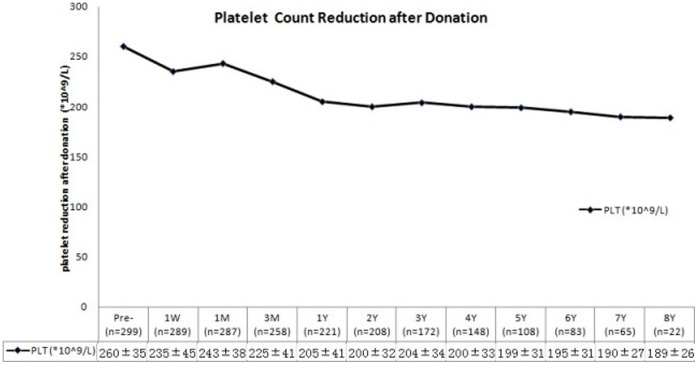
Changes in the platelet count (×10^9^/L) after donation at each follow-up time point.

There was no significant change in WBC or RBC count after donation compared with the pre-operative level; however, a persistent decrease in PLT count was observed one week after operation (Figure 2). Twenty-two cases were followed up for 8 years, and their mean PLT level (189×10^9^/L) remained lower than the pre-operative level (267×10^9^/L) but within the normal range (100×10^9^/L to 300×10^9^/L). There were two donors with PLT counts <100×10^9^/L. Although the PLT count showed an overall decreasing trend, a few cases presented a high PLT count (>300×10^9^/L).

### Health-related Quality of Life Assessment

Eight domains of health were used to measure the HRQoL using the SF-36 tool, including physical function, physical role, bodily pain, general health, vitality, social function, emotional role, and mental health during the last 12 mo. For the 252 validated questionnaire responses, the scores for the eight domains are listed in Table 3. The physical composite score and mental composite score were 54.3 and 50.6, respectively. Of these 252 donors, 98.4% (248) had returned to their previous level of social activity and an equal proportion to their pre-donation occupation. Moreover, 88.5% (223) of donors indicated their satisfaction with their present capacity to work, and 89.3% (225 cases) rated their present health status as good to excellent. However, 8.3% (21 cases) felt that the donation might have worsened their health in some way. A large majority of donors (96.0%, 242 cases) stated that they were completely satisfied with family life, except for 5 donors who reported a decrease in sexual activity. None of the donors regretted their decision to donate, and 99.2% of donors would make the donation again if it was required and feasible, except for two donors with grade III complications, whose recipients died. The psychosocial parameters after donation are shown in Table 3. A 46-year-old female donor developed slight psychiatric complications due to her recipient’s death. The syndrome was a depressive episode; however, the donor was successfully treated by our psychiatrist. Two other donors were diagnosed with an anxiety disorder, but both recovered to normal eventually.

**Table 3:** Health-Related Quality of Life and Psychological Symptoms after Donation.

SF-36 domains	Donors (252), mean±SD
**Health-related quality of life**	
Physical function	93.41±7.42
Role interference due to physical limitations	86.81±27.71
Bodily pain	78.17±20.22
General health	82.01±18.64
Vitality	68.32±21.21
Social functioning	83.22±15.21
Role interference due to emotional limitations	79.51±25.10
Mental health	75.41±18.35
**Psychological symptoms**	
Somatization	1.38±0.45
Obsessive-compulsive behavior	1.46±0.26
Interpersonal sensitivity	1.39±0.28
Depression	1.29±0.25
Anxiety	1.37±0.47
Hostility	1.57±0.47
Phobic anxiety	1.14±0.24
Paranoid ideation	1.27±0.15
Psychoticism	1.23±0.23

## Discussion

Donor safety must be the first priority during the entire process of living-donor liver transplantation, from the first day of evaluation through the entire follow-up period. It has become a more urgent issue with the rapidly rising frequency of LDLT due to the severe shortage of deceased donors in China. Donor mortality has been reported in several centers; therefore, more attention should be paid not only to the complications but also to laboratory test indicator changes and to the donor’s quality of life after donation. We have reported the living liver donors’ complication rate in our previous report [25], and here we evaluated the donors’ safety in three aspects: donor’s complications, laboratory parameter changes and health-related quality of life after donation.

Our study revealed a 25.3% donor morbidity rate using the Clavien 5-tier grading system after a median follow-up of 62 mo, which was similar to Kousoulas’ report [13] but much lower than Azoulay’s report of an overall complication rate of 47.3% [7]. The much higher complication rate in the Azoulay study was caused by the inclusion of only right lobe donations; intra-operative complications were also included. When the post-operative morbidity in the 42 first-stage cases was compared to the 34 second-stage cases, the overall complication rate in the first stage was much higher than that in the second stage (32.6% *vs* 19.9%); however, the distribution of grades of complication was similar. Ghobrial *et al* [8] reported that the most common complication was bacterial infection (12%). Our study also found that biliary complications were most common (9%) and mainly consisted of biliary leak. More complications took place in the right lobe donors than in the left lobe donors, and no complication was observed in the left lateral lobe donors. Overall, one case of biliary leak, two cases of wound infection and one case of abdominal bleeding requiring reoperation were observed after operation in the 42 left lobe donors. The overall complication rate in the left lobe donors was 9.5%, which was much lower than the 26.3% in the right lobe donors. In general, the potential risks associated with adult-to-adult LDLT are greater than with adult-to-child LDLT due to the extensive surgery and the smaller donor remnant in the former [26]. However, Kousoulas [13] indicated that there was no significant difference in the complication rates among the right lobe, left lobe and left lateral lobe donors after operation, that the severity of complications was comparable and that donor mortality and morbidity did not differ; however, right lobe donation was associated with prolonged hospital stay, increased frequency of blood transfusions and prolonged operation time compared with left and left lateral lobe donation.

New techniques may contribute to the improvement of outcomes of living donors. Innovations and refinements in the techniques of living-donor right hepatectomy have been made over the past decade. New technologies such as laparoscopic-assisted and minimal-access (MA) live-donor hepatectomy are effective and safe modalities for living-donor liver resections. In our liver transplantation center, laparoscopic-assisted hepatectomy has been used in 20 donors’ graft hepatectomies since June 2011. Laparoscopic-assisted and MA living-donor hepatectomy provide suitable grafts with early graft function and perioperative complication rates comparable to those achieved with standard open procedures [27]–[29]. Furthermore, our laparoscopic-assisted hepatectomy approach included an upper midline incision instead of the orthodox J-type incision, and the type and size of the abdominal incision affects the prognosis of the donor. Compared with the orthodox J-type incision, the upper midline incision with or without laparoscopic assistance can be used for LDLT with less pain and without impairing the safety, reproducibility, or efficacy of the procedure [30].

The decreased morbidity after January 2008 was strongly associated with the accumulating experience of our center. Specific training of the surgical team should be implemented in centers offering this procedure to enhance this learning curve [31]. Surgeon- and anesthesiologist-related factors are the most important complicating issues. The familiarization with this type of procedure and its peculiar characteristics, especially regarding the hemodynamics involved, may also contribute to the decreased complication rate. The surgeons, nurses and supporting staff should be aware of early warning signs that indicate potential complications in the donors. Surgeons and hospitals with high case volumes and specialist expertise produce better outcomes for complex surgical interventions [32], [33].

The aim of routinely monitoring the changes in laboratory test values was to evaluate the recovery of the donor and to identify complications earlier. In our analyses, we identified transient peaks of total bilirubinemia, AST, ALT and WBC count; however, most of the laboratory values approached baseline levels within 30 d after donation. In a 49-year-old donor with a peak total bilirubin of 248.4 µmol/L on the 4^th^ post-operative day with no biliary obstruction, as reflected by a GRWR of 0.69%, the small remnant liver may have contributed to the abnormally high bilirubinemia. The AST and ALT levels of donors were significantly increased postoperatively, which indicated hepatocellular damage. After comparing the changes in liver function after right hepatectomy between living donors and hepatic patients without cirrhosis, Li *et al* [17] found that the bilirubin levels of donors were significantly increased on the first and third postoperative days, which were much higher than the levels of the hepatic patients without cirrhosis; however, by the 7th postoperative day, the AST and ALT levels became similar between the two groups. Our attention should be focused on the persistently decreased PLT count observed in our donors. The etiology of the low PLT count is still unclear, although there are several possibilities [15]. First, some donors may have had elevated portal pressures. This could have been caused by inadequate regeneration of the hepatic remnant and a small remnant size, portal or hepatic venous insufficiency, or sinusoidal hyperperfusion, which could lead to increased portal pressure, splenomegaly, and a reduced PLT count. Second, thrombopoietin, the growth factor responsible for platelet production, is produced in the liver. All of the donors with transient abnormalities of liver function, blood cell counts and blood clotting function recovered quickly after operation, except for those with persistently low platelet counts.

The rapid growth of living-donor liver transplantation in China in recent years is attributable to the shortage of deceased-donor liver grafts. However, the donor of the LDLT is subjected to the risks of this surgical procedure, which may impose a considerable psychological burden on them. The donor is defined as a healthy person without significant medical or psychiatric problems; therefore, assessment of their psychosocial outcomes using questionnaires or scoring systems that compare donors to the general population can be difficult to interpret because selected donors generally have higher baseline scores than the general population [34]. Therefore, in our study, unlike in the report by Jin *et al* [35], no comparison was made between the donors and the general population.

After a surgery of such magnitude, some donors are likely to develop postoperative complications and persistent symptoms long after donation. Among these complications, biliary events appear to be pronounced. In our center, donor complications occurred in 25.3% of the donors. Although most donors eventually recover completely, some may unfortunately suffer from persistent symptoms, such as abdominal discomfort, fatigue, chronic pain and scar itching, possibly caused by surgical complications or by the surgery itself [7], [14], . The physical composite and mental composite scores were 54.3 and 50.6, respectively, which are similar to those reported by Bay *et al* [37]. Almost all of the donors went back to work, but only 88.3% were satisfied with their present status. This gap might have been caused by the donors’ recognition of their decreased involvement in vigorous activity. Some of them expressed increased anxiety and nervousness after surgery and worried about a lack of medical care from the doctor. Two donors stated their unwillingness to donate again because of severe complications and the death of their recipients, although 99.2% of donors indicated that they were willing to donate again.

In our liver and kidney transplantation center, all donors are assessed by our psychosocial team, who are also available for post-donation psychological support. Three donors were diagnosed with psychological disturbances by our psychiatrist. Luckily, all three donors recovered without the need for any drugs. Interestingly, all three of these donors were women. Jin *et al* indicated that females’ SF-36 scores were significantly lower than those of males, and these authors reached similar conclusions to ours in their psychological measures [35]. These data suggest that females suffer a relatively higher risk of developing psychological problems after LDLT. Consistent with our findings, successful recipient outcomes are very important to the psychological well-being of donors after operation [38]. In an investigation of factors that influence postoperative quality of life and psychological outcomes, Jin *et al* [35] analyzed 92 consecutive liver transplantation donors and demonstrated that gender, age, time after operation, recipient health condition and employment after donation influenced the donor’s postoperative quality of life. Meanwhile, the psychological measures indicated that donors were healthier than the general population in terms of obsessive-compulsive behavior, interpersonal sensitivity, phobic anxiety, and paranoid ideation. Furthermore, the mental component summary scale (MSC) of the SF-36 was significantly correlated with most of the symptom scores of the SCL-90-R.

In summary, an overall analysis of LDLT demonstrates a good safety profile for donors, with an overall morbidity of 25.3% and no mortalities. With advances in graft resection and other new techniques and ideas, significant improvements were observed in our complication rates. Donor safety considerations should include careful observation of laboratory test values. The cause of the persistent low platelet counts in our living donors warrants further evaluation. Additionally, postoperative quality of life and psychological outcomes should also be considered for the safety of the living donors. In conclusion, with careful donor selection and specialized patient care, low morbidity rates and satisfactory long-term recovery can be achieved after hepatectomy for living-donor liver transplantation, and liver donation is relatively safe for living donors. However, multi-center data should be collected and analyzed in future research in this field.
